# Sleep measures and cardiovascular disease in type 2 diabetes mellitus

**DOI:** 10.1016/j.clinme.2024.100021

**Published:** 2024-01-28

**Authors:** M. Engin

**Affiliations:** Bursa Yuksek Ihtisas Training and Research Hospital, Bursa, Turkey

I read the article by Magri *et al*^1^ entitled ‘Sleep measures and cardiovascular disease in type 2 diabetes mellitus’ with great interest. First of all, I congratulate the authors for their valuable contribution to the literature. However, I would like to discuss some points about type 2 diabetes mellitus (DM) and cardiovascular diseases.

Blood glucose control is a very important factor in the development of vascular diseases in type 2 DM patients. Currently, the HbA1c Variability Score is used as an important follow-up parameter in type 2 DM patients. In this regard, a recent study revealed that both low (<6.0 % [42 mmol/mol]) and high (≥8.0 % [64 mmol/mol]) glycemic control values were associated with increased all-cause mortality and DM-related macrovascular complications.^2^ Why did the authors not include glucose control parameters in the multivariate analyses in their current study? Did the study group consist of patients with very similar glycaemic control?

Blood values obtained from routine blood tests and indices obtained from these values can be used in the prognosis of many diseases.^3^ In their study, the authors determined the red blood cell distribution width (RDW) value as an independent predictor of macrovascular disease. What values (mean platelet volume, neutrophil, lymphocyte, platelet, white blood cell, neutrophil-lymphocyte ratio?) did they include in the analysis in their studies, other than RDW and C-reactive protein values?

Additionally, hyperlipidaemia is an important problem in Type 2 DM patients. The authors also detected hyperlipidaemia in 79.6 % of the patients in their study. What was the rate of antilipidemic drug use in the patients included in the study? In these patients, hypertriglyceridemia can be a particularly important problem. It is also believed to be associated with poor glucose control. In a recent study, its relationship with diabetes-related nephropathy was revealed.^4^ Was there a relationship between macrovascular and microvascular problems and triglyceride values in the authors’ study?
